# Hearing capacities and morphology of the auditory system in Serrasalmidae (Teleostei: Otophysi)

**DOI:** 10.1038/s41598-018-19812-1

**Published:** 2018-01-19

**Authors:** Geoffrey Mélotte, Eric Parmentier, Christian Michel, Anthony Herrel, Kelly Boyle

**Affiliations:** 1 Laboratoire de Morphologie Fonctionnelle et Evolutive, Institut de Chimie, Bât. B6c, Université de Liège, B-4000 Liège, Belgium; 2 Aquarium-Muséum, Département de Biologie, Ecologie et Evolution, Université de Liège, Institut de Zoologie, Bât I1, 22 quai Van Beneden, B - 4020 Liège, Belgium; 3 UMR 7179C.N.R.S./M.N.H.N., Département Adaptations du vivant, 55 Rue Buffon, Case Postale 55, 75005, Paris Cedex, 5 France; 4 Department of Marine Sciences, University of South Alabama, 5871 USA Drive North, Mobile, Alabama, 36688 USA; 5 Dauphin Island Sea Lab, 101 Bienville Boulevard, Dauphin Island, Alabama, 36528 USA

## Abstract

Like all otophysan fishes, serrasalmids (piranhas and relatives) possess a Weberian apparatus that improves their hearing capacities. We compared the hearing abilities among eight species of serrasalmids having different life-history traits: herbivorous vs. carnivorous and vocal vs. mute species. We also made 3D reconstructions of the auditory system to detect potential morphological variations associated with hearing ability. The hearing structures were similar in overall shape and position. All the species hear in the same frequency range and only slight differences were found in hearing thresholds. The eight species have their range of best hearing in the lower frequencies (50–900 Hz). In vocal serrasalmids, the range of best hearing covers the frequency spectrum of their sounds. However, the broad overlap in hearing thresholds among species having different life-history traits (herbivorous vs. carnivorous and vocal vs. non-vocal species) suggests that hearing ability is likely not related to the capacity to emit acoustic signals or to the diet, i.e. the ability to detect sounds is not associated with a given kind of food. The inner ear appears to be highly conservative in this group suggesting that it is shaped by phylogenetic history or by other kinds of constraints such as predator avoidance.

## Introduction

The ability to discriminate both biotic and abiotic sound sources is crucial for fishes^1^. They possess hearing structures that enable them to detect and identify different kinds of sounds in their environment, including conspecific calls, sounds produced by prey and predators, or abiotic cues that convey information on the environment^1–7^.

According to Ladich (2014)^8^, all fish species possess inner ears for sound detection but not all species possess sound generating mechanisms. This suggests that acoustic communication is not the primary constraint acting on the evolution of inner ears. Other life-history traits (diet, background noise, etc.) could correlate with differences in hearing ability. Serrasalmidae is an ideal family to examine co-variation in hearing and life-history traits because this family includes species that share different features: sound production vs. mute species and herbivorous vs. carnivorous species.

The Ostariophysan subgroup Otophysi is characterized by a series of modified elements (modifications of the first vertebrae, of the endo- and perilymphatic spaces of the inner ears and modifications of the swimbladder), called the Weberian apparatus, that connect the swimbladder to the inner ears^9–13^. The Weberian apparatus consists of paired symmetrical chains composed of one to four Weberian ossicles (tripus, intercalarium, scaphium and claustrum) that lie in a linear sequence from the swimbladder to the inner ear. Interossicular ligaments and articulation of the Weberian ossicles with their respective centra allow ossicles to hold in position on each side of the vertebral column^14^. Von Frisch (1938)^15^ demonstrated that these ossicular chains function in sound transmission. However, the claustrum is no longer considered as having an auditory function, at least in characiforms and siluriforms. In these groups, it has a protective function of the anterior portion of the neural canal^16,17^. When the swimbladder vibrates in a sound field, vibrations are transmitted to the ossicles and this motion is transmitted to the endolymphatic fluid causing sagitta (saccular otolith) displacements^18,19^.

Several studies have highlighted the important role of the Weberian apparatus in the improvement of hearing abilities, in terms of both hearing bandwidth and auditory sensitivity^12,20,21^. Ladich and Wysocki (2003)^22^ demonstrated that bilateral extirpation of the tripus in the otophysan *Carassius auratus*, goldfish, resulted in a hearing loss ranging from 7 dB at 100 Hz to 33 dB at 2 kHz. Yan *et al*. (2000)^23^ showed that the deflation of the swimbladder in the goldfish caused a drop in sensitivity by 33 to 55 dB depending on the frequency. However, otophysans do not exhibit a common morphology of the Weberian apparatus and swimbladder and this variation seems to have an impact on hearing ability. In catfishes, larger swimbladders and Weberian ossicles as well as greater numbers of ossicles are associated with improved hearing capacities^24^.

Our study focuses on the hearing abilities of eight species in the family Serrasalmidae. Four species are classified as leaf, fruit, or seed-eating^25–27^: *Piaractus brachypomus* (Cuvier, 1818); *Metynnis lippincottianus* (Cope, 1870); *Myloplus rubripinnis* (Müller and Troschel, 1844) and *Myleus schomburgkii* (Jardine, 1841). The other four, *Serrasalmus elongatus* Kner, 1858; *Serrasalmus spilopleura* Kner, 1858; *Pygocentrus nattereri* Kner, 1858 and *Pygocentrus piraya* (Cuvier, 1819), are classified as carnivorous piranhas^28,29^. Moreover, we note that five of these species are known to produce sounds. *Serrasalmus elongatus*, *S. spilopleura* and *Py. nattereri* emit low frequency sounds by contracting fast sonic muscles attached to the anterior chamber of the swimbladder^30–32^. *Piaractus brachypomus* and *Pygocentrus piraya* are also able to produce low frequency calls (unpublished data). The first goal of our study was to determine hearing capacities of these eight species and to investigate co-variation in hearing abilities and life-history traits (carnivorous vs. herbivorous and vocal vs. non-vocal species). The second objective was to compare the morphology of hearing structures among the species and to evaluate if morphological variation may explain differences in hearing abilities. Our hypothesis is that vocal species as well as carnivorous piranhas would have better hearing abilities to optimize intraspecific acoustic communication and to localize their living prey, respectively.

## Results

### Hearing capacities

**Figure 1:**
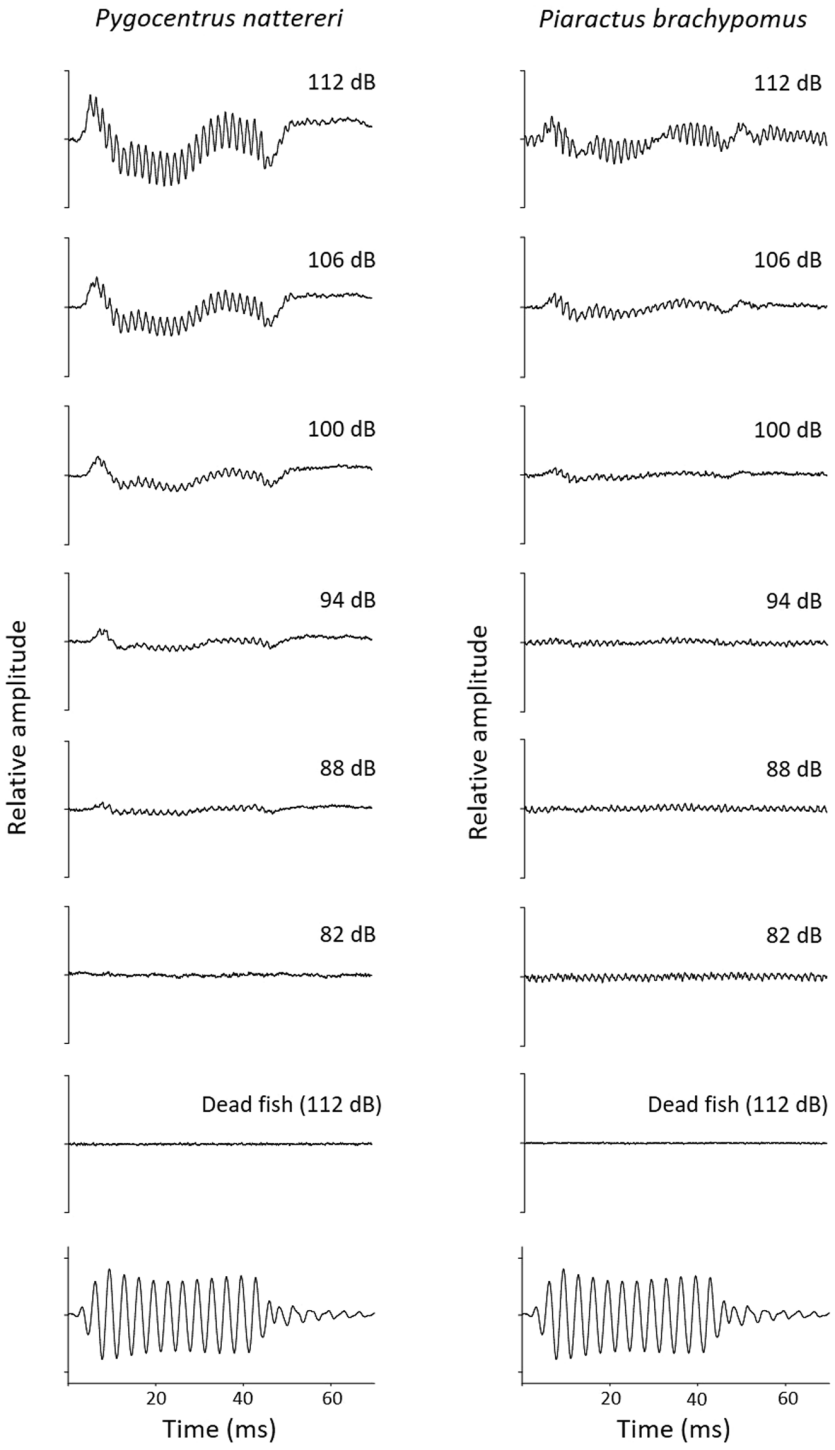
Example of auditory evoked potentials (AEP) measured at 300 Hz from one individual of *Pygocentrus nattereri* and one individual of *Piaractus brachypomus*. The traces show the averaged evoked response at six different stimulus intensities. The bottom trace shows the stimulus waveform recorded by the hydrophone at the position of the fish head. The AEP thresholds (i.e. the lowest sound pressure level to show a definitive response) for these individuals were 88 dB re 1 µPa in *Py. nattereri* and 100 dB re 1 µPa in *Pi. brachypomus*. There was no response from any dead specimen.

Auditory evoked potentials (AEP) were obtained for the eight serrasalmid species. AEP traces were similar in shape within a given frequency across all individuals (Fig. 1). Waveforms produced in response to stimulus presentation decreased in magnitude as the sound pressure level decreased, and were used to determine AEP thresholds of the eight species (Fig. 1).

**Figure 2:**
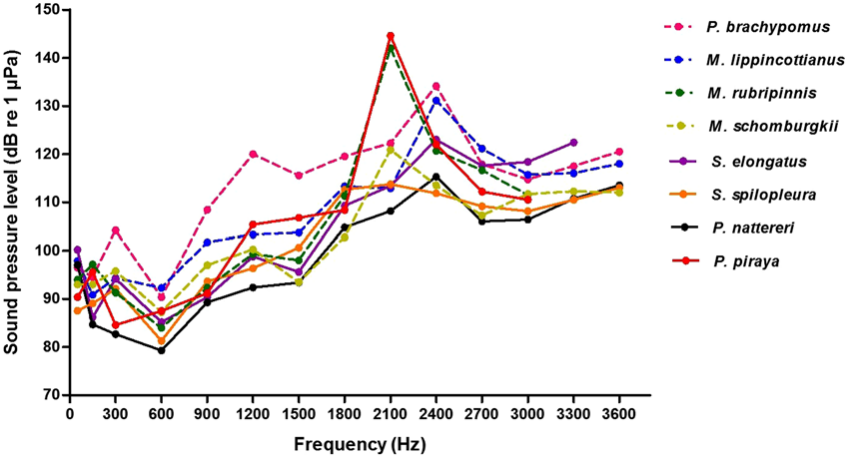
Audiograms of *Piaractus brachypomus*, *Metynnis lippincottianus*, *Myloplus rubripinnis*, *Myleus schomburgkii*, *Serrasalmus elongatus*, *Serrasalmus spilopleura,* *Pygocentrus nattereri* and *Pygocentrus piraya*. For greater clarity, standard deviations are not represented.

Five species out of eight were able to detect frequencies ranging from 50 to 3600 Hz (Fig. 2). *Serrasalmus elongatus* detects frequencies up to 3300 Hz, whereas the frequency range of *Myloplus rubripinnis* and *Pygocentrus piraya* extends up to 3000 Hz. The eight serrasalmid species were most sensitive at the lower frequencies tested, between about 50 and 900 Hz (Fig. 2). The eight species are characterized by the presence of a peak (at 2100 Hz or 2400 Hz according to the species) in their hearing curve. This high-frequency peak is particularly marked in *M. rubripinnis* and *Py. piraya* (Fig. 2, Table 1). Hearing thresholds of all species increase from 600 Hz to this peak (at 2100 Hz or 2400 Hz) and then decrease in higher frequencies (from 2400 Hz to 3000 Hz). Statistical tests (nested ANOVA, *P* < 0.001) revealed a significant species effect on AEP thresholds (Table 2). *Post hoc* tests (Tukey’s HSD) showed that, even if significant differences in auditory thresholds exist among certain species at certain frequencies, there is no clear distinction among the species over the entire frequency range (see Supplementary Table S1 for a detailed comparison among species). The two species that differed most in terms of auditory sensitivity are *Pi. brachypomus* and *Py. nattereri*. *Pygocentrus nattereri* was significantly more sensitive than *Pi. brachypomus* at 300 Hz, 900 Hz, 1200 Hz, 1500 Hz, 1800 Hz, 2100 Hz and 2400 Hz (*P* ≤ 0.001). At 1200 Hz, the difference of sensitivity exceeded 27 dB SPL (Table 1). The other serrasalmids possessed intermediate auditory thresholds between these two species for the majority of the frequencies tested.

**Table 1:** Hearing thresholds of *Piaractus brachypomus, Metynnis lippincottianus, Myloplus rubripinnis, Myleus schomburgkii, Serrasalmus elongatus, Serrasalmus spilopleura, Pygocentrus nattereri* and *Pygocentrus piraya*.

Frequency (Hz)	*Piaractus brachypomus*		*Metynnis lippincottianus*		*Myloplus rubripinnis*		*Myleus schomburgkii*		*Serrasalmus elongatus*		*Serrasalmus spilopleura*		*Pygocentrus nattereri*		*Pygocentrus piraya*	
	M ± SD	N	M ± SD	N	M ± SD	N	M ± SD	N	M ± SD	N	M ± SD	N	M ± SD	N	M ± SD	N
50	96.5 ± 10	10	97.9 ± 9.7	10	94.0 ± 9.2	3	93 ± 4.5	8	100.2 ± 4.3	5	87.6 ± 3.2	7	97.1 ± 8.2	10	90.4 ± 4.6	5
150	94.6 ± 5.8	10	90.9 ± 3.5	10	97.2 ± 6.8	3	93 ± 4.2	8	86.2 ± 6.2	5	89.1 ± 3.2	7	84.7 ± 5.9	10	95.6 ± 12.4	5
300	104.2 ± 6.8	10	94.4 ± 4.1	10	91.3 ± 3.3	3	95.8 ± 7.4	8	94.2 ± 6.1	5	92.1 ± 6.4	7	82.7 ± 11.5	10	84.6 ± 7.9	5
600	90.4 ± 3.9	10	92.3 ± 5.9	10	84.0 ± 6.0	3	87.3 ± 2.1	8	85.2 ± 7	5	81.3 ± 4.5	7	79.3 ± 6.2	10	87.5 ± 4.8	5
900	108.5 ± 5.9	10	101.8 ± 8.7	10	92.3 ± 3.1	3	97 ± 3.7	8	90.4 ± 4.3	5	93.6 ± 4.1	7	89.3 ± 10.2	10	91.2 ± 11.1	5
1200	120 ± 4.2	10	103.3 ± 4.4	10	99.3 ± 1.2	3	100.3 ± 4.1	8	98.8 ± 5.6	5	96.4 ± 5.4	7	92.4 ± 4	10	105.4 ± 5.8	5
1500	115.6 ± 5.8	10	103.7 ± 4.5	10	98.0 ± 5.2	3	93.5 ± 4.8	8	95.6 ± 7	5	100.6 ± 5.4	7	93.4 ± 4.5	10	106.8 ± 5.2	5
1800	119.6 ± 5.4	10	113.3 ± 3.7	10	111.3 ± 3.2	3	102.8 ± 4.9	8	109.3 ± 4.4	5	112.6 ± 4.1	7	104.8 ± 9	10	108.4 ± 4.8	5
2100	122.3 ± 4.3	10	112.9 ± 4.7	10	142.0 ± 6.0	3	120.9 ± 7.7	8	113.4 ± 8.1	5	113.7 ± 6.7	7	108.2 ± 6.6	10	144.6 ± 10	5
2400	134.1 ± 4.1	7	131.1 ± 6.9	7	120.7 ± 1.2	3	113.5 ± 4.5	8	123 ± 7.1	5	111.9 ± 2.3	7	115.3 ± 4.7	10	122 ± 7.3	5
2700	117.9 ± 5.6	4	121.1 ± 2.4	8	116.7 ± 6.7	3	107.3 ± 2.8	8	117.6 ± 10.7	5	109.1 ± 4.1	7	106 ± 6.9	10	112.2 ± 8.8	5
3000	114.8 ± 7.2	8	115.7 ± 5.5	9	111.5	1	111.7 ± 3.6	8	118.4 ± 6.2	5	108.1 ± 4.7	7	106.4 ± 7.7	10	110.5 ± 4.9	2
3300	117.5 ± 7.8	2	116 ± 5.4	6	—	—	112.3 ± 3.8	8	122.4 ± 5	5	110.5 ± 4.5	7	110.7 ± 5.1	10	—	—
3600	120.5	1	118 ± 0	2	—	—	112 ± 1	5	—	—	113 ± 0	4	113.5 ± 3	4	—	—

Hearing thresholds (dB re 1 µPa) are expressed as mean ± standard deviation; N, number of individuals responding to the stimulus.

**Table 2:** Nested ANOVA using ‘auditory sensitivity’ as a dependent variable and ‘species’ and ‘frequency’ as fixed factors (categorical predictors). Significant *P*-values are in bold.

Source	d.f.	MC	*F*	*P*
Species	7	1539	42.1	**<0.001**
Frequency x Species	99	1077	29.4	**<0.001**
Error	621	37		

**Figure 3:**
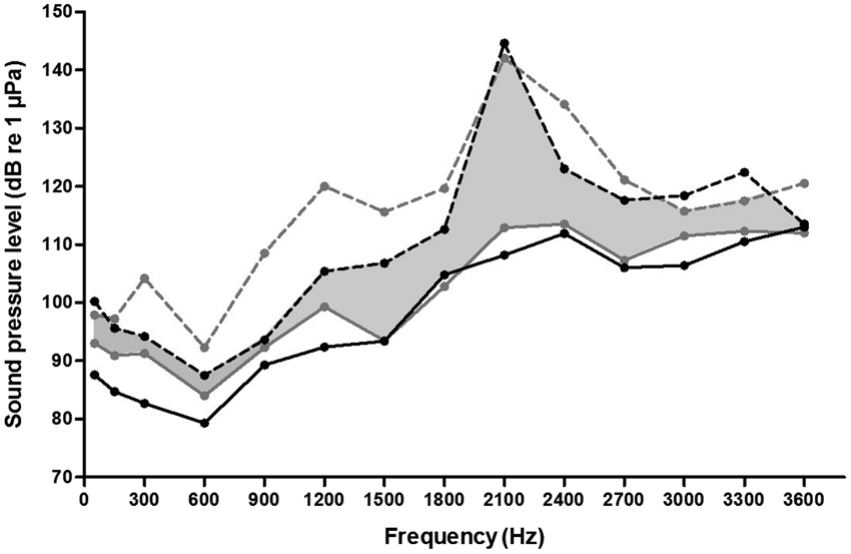
Hearing curves of minimum and maximum sensitivity for herbivorous (*Piaractus brachypomus*, *Metynnis lippincottianus*, *Myloplus rubripinnis* and *Myleus schomburgkii*) and carnivorous species (*Serrasalmus elongatus*, *Serrasalmus spilopleura*, *Pygocentrus nattereri* and *Pygocentrus piraya*). The area between the grey lines contains hearing thresholds of herbivorous species. The area between the black lines contains hearing thresholds of carnivorous species. The shaded area corresponds to the range of hearing thresholds overlapping between the two groups. Hearing curves of minimum sensitivity for herbivorous species (dashed grey line), maximum sensitivity for herbivorous species (solid grey line), minimum sensitivity for carnivorous species (dashed black line), maximum sensitivity for carnivorous species (solid black line).

**Figure 4:**
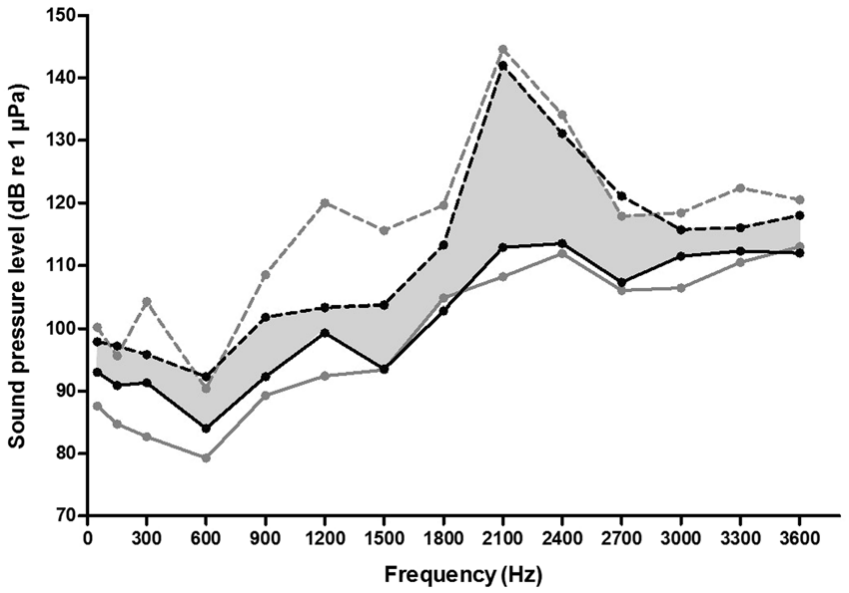
Hearing curves of minimum and maximum sensitivity for vocal (*Piaractus brachypomus*, *Serrasalmus elongatus*, *Serrasalmus spilopleura*, *Pygocentrus nattereri* and *Pygocentrus piraya*) and non-vocal species (*Metynnis lippincottianus*, *Myloplus rubripinnis* and *Myleus schomburgkii*). The area between the grey lines contains hearing thresholds of vocal species. The area between the black lines contains hearing thresholds of non-vocal species. The shaded area corresponds to the range of hearing thresholds overlapping between the two groups. Hearing curves of minimum sensitivity for vocal species (dashed grey line), maximum sensitivity for vocal species (solid grey line), minimum sensitivity for non-vocal species (dashed black line), maximum sensitivity for non-vocal species (solid black line).

The hearing range among herbivores (*Pi. brachypomus*, *M. lippincottianus*, *M. rubripinnis* and *M. schomburgkii*) and carnivores (*S. elongatus*, *S. spilopleura*, *Py. nattereri* and *Py. piraya*) largely overlapped (Fig. 3). Similarly, the hearing range of vocal (*Pi. brachypomus*, *S. elongatus*, *S. spilopleura*, *Py. nattereri* and *Py. piraya*) and non-vocal species (*M. lippincottianus*, *M. rubripinnis* and *M. schomburgkii*) also largely overlapped (Fig. 4). For the eight serrasalmid species, linear regressions were performed between fish SL and hearing thresholds at each frequency tested. There were no significant correlations between fish size and hearing sensitivity for any species (see Supplementary Table S2).

### Morphology

**Figure 5:**
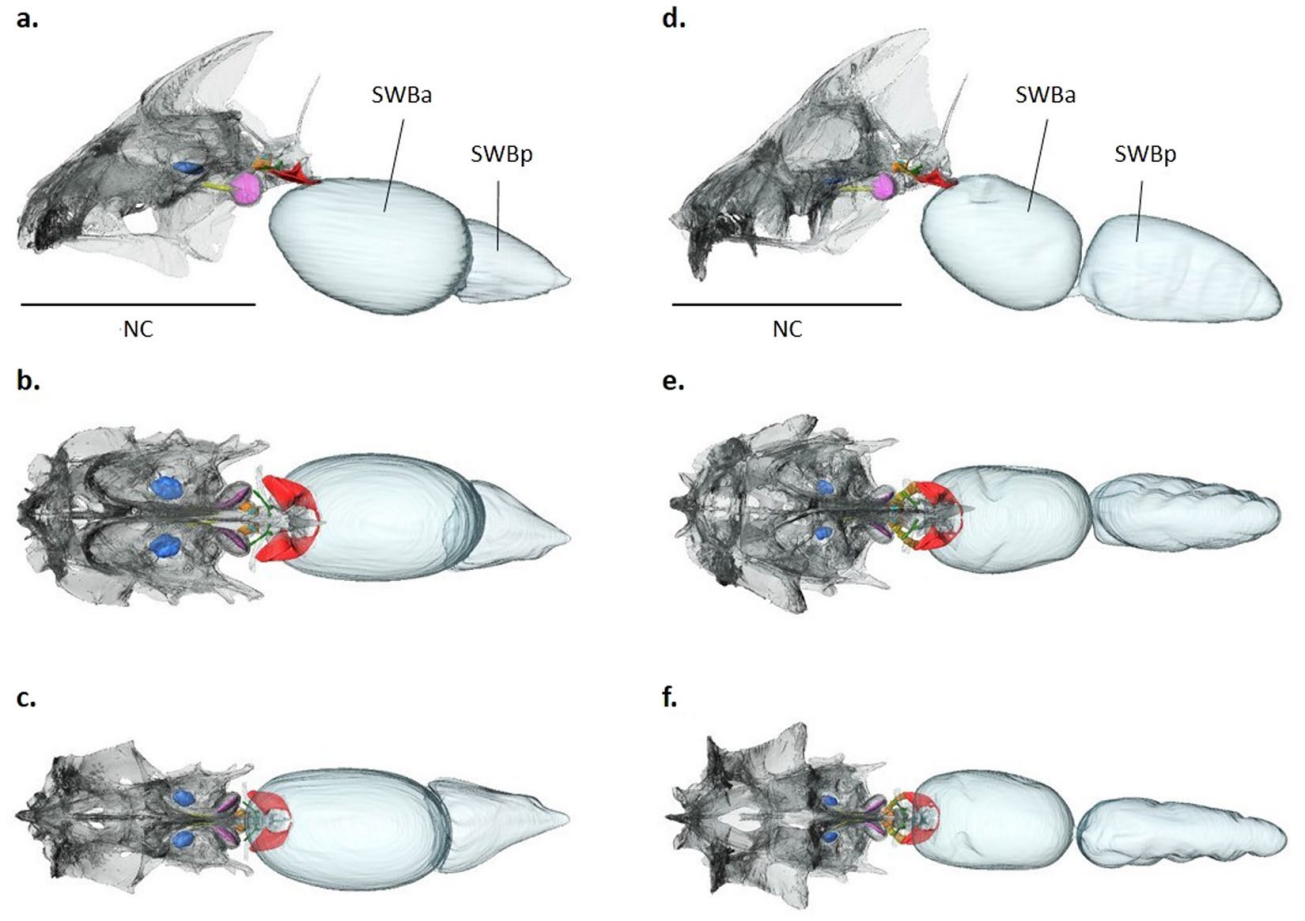
Three-dimensional reconstruction of the neurocranium, the swimbladder, otoliths and Weberian ossicles in *Pygocentrus nattereri* and *Piaractus brachypomus*. (**a**) Left lateral, (**b**) dorsal and (**c**) ventral views of the reconstructed anterior part of *Py. nattereri*. (**d**) Left lateral, (**e**) dorsal and (**f**) ventral views of the reconstructed anterior part of *Pi. brachypomus*. The 3D reconstructions are based on µCT scans. Note that the interossicular ligaments between the Weberian ossicles are visible on the reconstructions of *Pi. brachypomus*. Lapillus (blue), sagitta (yellow), asteriscus (fuchsia), tripus (red), intercalarium (green), scaphium (orange) and claustrum (sky blue). NC, neurocranium; SWBa, anterior part of the swimbladder; SWBp, posterior part of the swimbladder. Scale data: neurocranium length is 32 mm and 37.7 mm in *Py. nattereri* and *Pi. brachypomus*, respectively.

**Figure 6:**
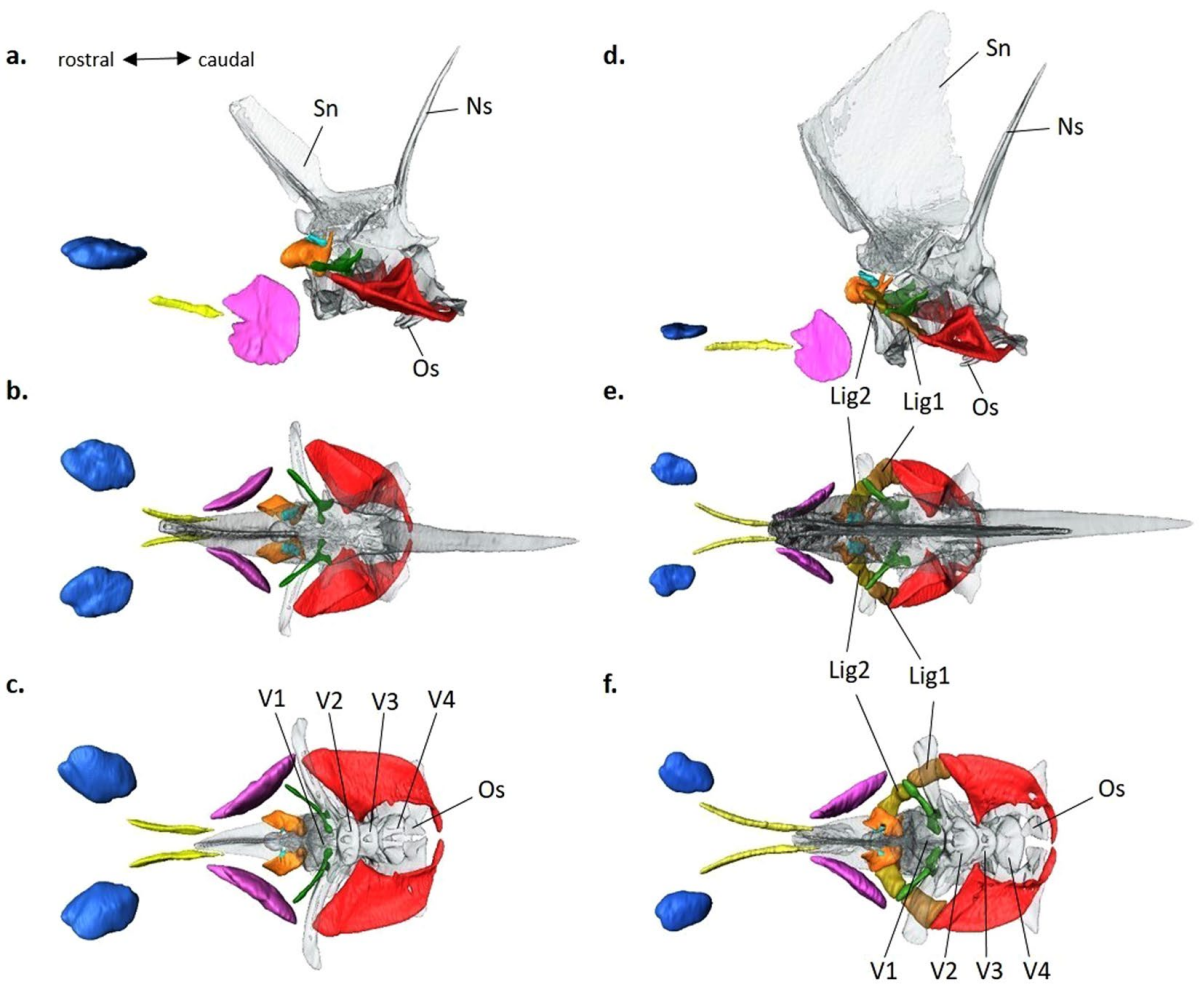
Three-dimensional reconstruction of otoliths, Weberian ossicles and associated bones in *Pygocentrus nattereri* and *Piaractus brachypomus*. (**a**) Left lateral, (**b**) dorsal and (**c**) ventral views of the reconstructed structures in *Py. nattereri*. (**d**) Left lateral, (**e**) dorsal and (**f**) ventral views of the reconstructed structures in *Pi. brachypomus*. The 3D reconstructions are based on µCT scans. Note that (1) right otoliths were not represented in Fig. 6a,d for greater clarity; (2) the interossicular ligaments between the Weberian ossicles are visible on the reconstructions of *Pi. brachypomus*. Lapillus (blue), sagitta (yellow), asteriscus (fuchsia), tripus (red), intercalarium (green), scaphium (orange) and claustrum (sky blue). Lig1, interossicular ligament 1; Lig2, interossicular ligament 2; Ns, neural spine; Os, os suspensorium; Sn, supraneural; V1–4, vertebrae 1-4. Scale data: lapillus length is 3.7 mm and 2.7 mm in *Py. nattereri* and *Pi. brachypomus*, respectively.

The structures of interest (otoliths, Weberian ossicles and the anterior swimbladder chamber) were described by means of three-dimensional reconstructions based on CT scans. Two species, *Pygocentrus nattereri* and *Piaractus brachypomus*, were selected for illustration because they show the greatest differences in hearing sensitivity (Figs 5 and 6).

The position and overall shape of the auditory structures were largely similar in the six species investigated (*Py. nattereri*, *Pi. brachypomus*, *M. rubripinnis*, *M. schomburgkii, S. elongatus* and *M. lippincottianus*). The asteriscus (lagenar otolith) was the most voluminous otolith, with a mean value between 58.7% and 85.7% of total otolith volume (Figs 5, 6). The lapillus (utricular otolith) was the second most voluminous (mean value between 9.5% and 39% of total otolith volume) and the sagitta (saccular otolith) was least voluminous (mean value between 2.3% and 6.9% of the total otolith volume; see Supplementary Table S3 for detailed morphological data of otoliths). *Pygocentrus nattereri* possessed the least voluminous sagittae (2.3% of the total otolith volume) and *Pi. brachypomus* the most voluminous (6.9% of the total otolith volume). The overall shape and location of the three pairs of otoliths in the neurocranium were similar in the six species (Figs 5, 6). The semicircular canals were not visible on CT scans.

The number of ossicles in the Weberian apparatus was the same in the six species (Figs 5, 6). Each species possessed a tripus, an intercalarium, a scaphium and a claustrum. The tripus was the largest Weberian ossicle in the six species, with a mean value between 73.5% and 81.8% of the total Weberian ossicle volume. As revealed by the reconstructions based on CT scans, the tripus contacts the anterior part of the swimbladder (Fig. 5a,d). *Pygocentrus nattereri* was characterized by the most voluminous anterior swimbladder (88.6% of the total swimbladder volume; see Supplementary Table S4 for detailed morphological data of Weberian ossicles and swimbladder). Volume and overall aspect and form of the Weberian ossicles were similar in the six serrasalmid species. However, some features presented slight differences among the species investigated. For example, *Piaractus brachypomus* has a tripus that is not as a laterally expanded compared to the other species. *Piaractus brachypomus* also has a wider lateral process on the intercalarium than the other species. For the scaphium, there is variation in the shape of the anterior margin of the concha scaphium (it is more rounded and extended in the piranhas and *Metynnis*) and the ascending process is shorter in the piranha species (*Py. nattereri* and *S. elongatus*).

For each hearing structure (otoliths, Weberian ossicles and the anterior swimbladder), linear regressions were performed between the averaged relative morphological measurements of the structure and hearing thresholds at each frequency tested. No clear relationship between the morphology of these structures and hearing sensitivity was observed in serrasalmids (see Supplementary Table S5 for detailed results of the regressions). When we looked for trends between morphology and hearing sensitivity at three larger frequency bands (50–1200 Hz, 1500–2400 Hz and 2700–3600 Hz), we observed an association between sagittal morphology and hearing sensitivity in high frequencies (2700–3600 Hz). The larger the sagitta, the lower the hearing sensitivity for high frequencies (relative length: R² = 0.781, *P* = 0.0195; relative volume: R² = 0.739, *P* = 0.028; relative surface area: R² = 0.914, *P* = 0.003; see Supplementary Table S6 for detailed results of the regressions).

## Discussion

The Weberian apparatus is well-known to improve hearing abilities of otophysan fishes, in terms of both hearing bandwidth and hearing sensitivity^20,21^. The eight serrasalmid species investigated in our study were able to detect frequencies up to at least 3000 Hz which is common to Otophysi^19,22,33,34^. Surprisingly, all species were characterized by a low sensitivity peak in their audiogram (around 2400 Hz) followed by an increase in sensitivity in higher frequencies. Comparisons of AEP audiograms showed few differences in mean hearing thresholds among the studied species.

All the species had their best hearing sensitivity in the low frequencies (50–900 Hz). Stabentheiner (1988)^35^ previously determined the hearing thresholds of *Pygocentrus nattereri* by means of a behavioral method. He found that the highest sensitivity was between 100 and 600 Hz. He noted that the range of best hearing covers the frequency spectrum of drumming sounds produced by this species. This is also the case for the vocal species investigated in our study. Sounds emitted by *S. elongatus*, *S. spilopleura*, *Py. nattereri* and *Py. piraya* have a fundamental frequency between 100 and 200 Hz and contain harmonics^30–32^, whereas sounds produced by *Pi. brachypomus* have a dominant frequency around 80 Hz without harmonics (personal observations; unpublished data). We infer that vocal serrasalmid species used structures to produce sounds within the hearing range in which they hear best. However, the hearing thresholds of non-vocal species are similar to the hearing thresholds of vocal species (Fig. 4). This indicates that hearing capacities in serrasalmids are not related to the ability to produce sounds. Although previous comparisons did not concern species of the same taxa, it was previously hypothesized that selective pressures might be related to other relevant acoustic cues, such as predator and food detection in quiet freshwater habitats^2,36^. The auditory system of both vocal and non-vocal fish species from different taxa can possess high temporal resolution abilities, reinforcing the idea that the primary function of acute hearing may not be acoustic communication^37^. As in Serrasalmidae, Ladich and Popper (2001)^38^ showed that three species of acanthomorph labyrinth fishes (gouramis) differing widely in their abilities to produce sounds possessed similar auditory sensitivity and similar sensory epithelia morphology.

Food and prey detection could also act as selective pressures on hearing abilities of fishes^2,36^. A splashing noise emitted by a hurt fish swimming near the water surface is known to attract carnivorous piranhas and to motivate them to attack^39^. Herbivorous serrasalmids primarily eat seeds and fruits^25–27^. Consequently one could expect that their hearing abilities, in addition to their lateral line system, might allow them to detect these items falling into the water. However, the range of hearing thresholds of herbivorous and carnivorous species strongly overlapped (Fig. 3) and the range is not obviously related to the detection of food items.

The number of ossicles comprising the Weberian apparatus was the same in each species and the overall shape and position of these ossicles were quite comparable, despite slight morphological variation among the species. The otoliths were also similar in overall shape and position. We found an association between sagittal morphology and hearing sensitivity at the highest frequencies tested (2700–3600 Hz). Small sagittae are correlated with low hearing thresholds (i.e. better hearing abilities). Chardon and Vandewalle (1997)^12^ hypothesized that smaller sagittae in otophysans would reduce inertia to detect the small fluid movements transduced from the Weberian ossicles. Based on this hypothesis, we would expect lower mass sagittae to be associated with high hearing sensitivity. In fact, species with the best hearing sensitivity (in the frequency range 2700–3600 Hz) in our study possessed the smallest sagittae. However, further studies using microphonics, an electrophysiological recording technique, are needed to test experimentally the influence of sagittal morphology on high frequencies sensitivity^40,41^. It is also worth mentioning that the majority of significant hearing differences among serrasalmids were observed in the frequency range 900–2400 Hz. These hearing differences were not correlated with observed morphological variation.

The slight differences in hearing sensitivity among the species investigated in our study could be due to more subtle morphological variation. For example, the slight morphological disparities observed in tripus shape or in the length of ossicles processes could influence hearing sensitivity. Moreover, the orientation patterns of ciliary bundles of the sensory hair cells on the maculae (sensory epithelia associated with the otoliths) could also have an impact on the acoustic abilities in fishes^42–44^. Unfortunately, these last structures were not visible on the µCT scans and were not reconstructed.

Morphological variation of the Weberian apparatus within a single fish family has rarely been studied. Within the otophysan Cypriniformes, the morphology of the Weberian apparatus is generally diagnostic for each family and only slight interspecific variations of the Weberian apparatus have been documented. These modifications comprised subtle changes in shape and length of Weberian ossicle processes (e.g. Bird and Hernandez)^45^. Contrary to Cypriniformes, the diversity in swimbladder and Weberian apparatus morphology in the otophysan Siluriformes (catfishes) is high. Lechner and Ladich (2008)^24^ showed that larger swimbladders and ossicles as well as higher ossicle number (i.e. less evolutionary loss of ossicles) improve hearing at higher frequencies in catfishes. The relative lengths of swimbladders and of ossicular chains were correlated with hearing sensitivity above 1 and 2 kHz, respectively, whereas the number of ossicles affected hearing at 4 and 5 kHz. In our study, the number and the morphology of Weberian ossicles were conserved among the species and did not have an obvious impact on hearing abilities.

The size ranges of the different species indicate that the majority of specimens were juveniles or subadults. Hearing range and auditory sensitivity are known to increase along with the development of the auditory structures^20,21^. In the goldfish *Carassius auratus*, the Weberian apparatus reaches the adult condition at 25 mm length^46^. In the African bullhead catfish *Lophiobagrus cyclurus*, Weberian ossicles and ligaments are fully developed at 24 mm SL^21^, whereas ossicles are fully developed at 15 mm TL in the air-breathing catfish, *Clarias gariepinnus*^47^. According to the size of serrasalmid specimens used in AEP experiments, they all have a completely developed Weberian apparatus meaning the ear is fully functional. Although it is not always the case^10,20,48^, hearing sensitivity could potentially change with fish size, as in the catfish *L. cyclurus* that showed significant frequency-dependent change in hearing thresholds with size but no change in the range of detectable frequencies.

It is worth mentioning that there are a number of rheophilic serrasalmid species which were not included in this study^49,50^. Some authors hypothesized that hearing in fish may be adapted to the ambient noise of the fish’s habitat^8,51^. Since rheophilic species live in noisy rapids, it would be quite interesting to determine if their hearing abilities and the morphology of their auditory system vary compared to serrasalmid species living in quieter freshwater habitats.

In serrasalmids, the broad overlap in hearing ability among species having different life-history traits (vocal vs. non-vocal and herbivorous vs. carnivorous) suggests that hearing capacities are likely not related to the ability to produce sounds nor to food detection. The morphology of otoliths and Weberian apparatus is largely similar in the species investigated and does not explain obviously differences in hearing abilities. The slight differences in hearing thresholds among the species could be related to more subtle differences in shape and functional performance of the different Weberian ossicles or to variations in the shape of maculae or in the orientation patterns of ciliary hair cells on sensory epithelia.

## Methods

### Fish collection

Ten *Piaractus brachypomus* (69 to 95 mm SL), 10 *Metynnis lippincottianus* (76 to 87 mm SL), 3 *Myloplus rubripinnis* (98 to 108 mm SL), 8 *Myleus schomburgkii* (55 to 61 mm SL), 5 *Serrasalmus elongatus* (66 to 79 mm SL), 7 *Serrasalmus spilopleura* (75 to 105 mm SL), 10 *Pygocentrus nattereri* (64 to 70 mm SL) and 5 *Pygocentrus piraya* (96 to 112 mm SL) were purchased in the aquarium trade. They were housed in freshwater aquaria at 26 ± 1 °C and were maintained on a 12 h light/dark cycle. The tanks were equipped with external filters, internal heaters and bubblers for aeration. Herbivorous fishes were fed granule food (JBL Novo Tab), whereas flesh-eating species were fed mussels, three times a week. All procedures were approved by the ethical commission of the University of Liège (ethics case 1532).

### Hearing capacities

#### AEP thresholds measurement: experimental setup

Hearing thresholds were measured using the AEP technique^52,53^. This technique records summation of acoustically evoked neuronal responses along the ascending auditory pathways from peripheral hearing end organs to the brain region^53^. Presence or absence of a response to acoustic stimuli of different frequencies and intensities allows the determination of AEP thresholds. The experimental setup was similar to that used by Parmentier *et al*. (2009)^54^ and Colleye *et al*. (2016)^55^.

No anaesthetic was used to restrain the fish during the AEP recordings. However, each fish was immobilized in a custom-made harness in order to prevent body movements while allowing normal respiration. The harness was closed dorsally and caudally with clamps attached to a steel frame. Three subdermal stainless steel needle electrodes (Rochester Electro-Medical, Lutz, FL, USA) were used to record electric signals. The recording electrode was inserted approximately 1 mm into the head, over the otic region. The reference electrode was placed into the fish’s epaxial musculature, whereas the ground electrode was in the water in close vicinity to the fish. Fish were suspended 10 cm below the water surface in a steel tube (1.15 m high, 22 cm diameter, 0.7 cm thickness) closed at the bottom with a square steel plate (40 × 40 cm). The tube was oriented vertically and filled with freshwater of approximately 26 °C up to a height of 1.12 m. An underwater loudspeaker (UW-30, Lubell Labs, Columbus, OH, USA) was placed at the bottom of the steel tube. The entire setup was enclosed in a soundproof chamber.

#### Stimulus generation and AEP recordings

A Tucker-Davies Technologies (TDT, Alachua, FL, USA) AEP workstation was used to generate sound stimuli and record AEP waveforms. TDT SigGen software was used to create sound stimuli with an RP2.1 enhanced real-time processor, a PA5 programmable attenuator to control sound level, and a power amplifier before being sent to and emitted by the underwater speaker. Sound stimuli were tone bursts of 50 ms in duration gated with a Hanning window. The phase of the tone was alternated between presentations to minimize electrical artefacts from the recordings. Fifteen frequencies were presented to each fish: 50, 150, 300, 600, 900, 1200, 1500, 1800, 2100, 2400, 2700, 3000, 3300, 3600 and 4000 Hz. At each frequency, sound levels were presented up to 164 dB re 1 µPa and were attenuated in 6 dB steps until a threshold level was determined. Evoked potentials recorded by the electrode were amplified (TDT HS4-DB4 amplifier, 10,000 gain), connected to an RP2.1 enhanced real-time processor, routed into the computer and averaged by BioSig software. At each frequency and for each sound level, the signal was presented 500 times. Sound levels of the acoustic stimuli were calibrated with a Brüel and Kjær 8101 hydrophone (Nærum, Denmark; sensitivity -164 dB re 1 V/µPa; bandwidth 0.1 Hz to 200 kHz) placed in the steel tube at the previous position of the fish’s head. The hydrophone was connected to a calibrated Brüel and Kjær 2610 amplifier that gave the absolute pressure level of sound stimuli. Evoked responses were averaged and power spectra were calculated using a 4096-point Fast Fourier Transform (FFT). The spectra were analysed for the presence of peaks at twice the stimulus frequency with heights at least 3 dB above the background level. For each frequency, the lowest sound level at which such peaks were present was defined as the auditory threshold for that frequency. Dead specimens of the eight species were also tested to confirm that recorded AEP traces were not artefacts. No responses were recorded with dead fishes (Fig. 1).

In addition to the hearing curves of each species, we also traced the hearing curves of maximum and minimum sensitivity for herbivorous (*Pi. brachypomus*, *M. lippincottianus*, *M. rubripinnis* and *M. schomburgkii*) and carnivorous species (*S. elongatus*, *S. spilopleura*, *Py. nattereri* and *Py. piraya*), as well as for vocal (*Pi. brachypomus*, *S. elongatus*, *S. spilopleura*, *Py. nattereri* and *Py. piraya*) and non-vocal species (*M. lippincottianus*, *M. rubripinnis* and *M. schomburgkii*). To achieve this, we selected the highest and lowest hearing thresholds among the species of each group (herbivorous vs. carnivorous, vocal vs. non-vocal) at each frequency tested.

### Morphology

The morphology of the auditory system of three *Pygocentrus nattereri* (120 mm, 134 mm and 155 mm in SL), three *Piaractus brachypomus* (131 mm, 139 mm and 168 mm in SL), two *Myloplus rubripinnis* (136 mm and 140 mm in SL), two *Myleus schomburgkii* (242 mm and 252 mm in SL), four *Serrasalmus elongatus* (63 mm, 88 mm, 119 mm and 152 mm in SL), and four *Metynnis lippincottianus* (96 mm, 102 mm, 109 mm and 112 mm in SL) was investigated using computed tomography (CT). These specimens were scanned at the National Museum of Natural History in Paris with a µCT scanner (v|tome|x 240 L, GE Sensing & Inspection Technologies phoenix|x-ray). The imaging system was set at 70 kV and specimens were scanned at an isotropic voxel size between 37.1 and 99.9 µm. Volume and surface rendering was performed with AMIRA 6.3 and AVIZO (VSG, Fei Company). Morphological measurements were performed with AVIZO. For each specimen, we measured the volume of the three Weberian ossicles having an auditory function (tripus, intercalarium and scaphium), the three otoliths (lapillus, sagitta and asteriscus) and the anterior part of the swimbladder. We also measured the surface area of each otolith as well as the length of the sagitta. In addition, the ratios of the volume of each Weberian ossicle to the total Weberian ossicle volume were calculated. The ratios of the volume (and surface) of each otolith to the total otolith volume (and total otolith surface) were also calculated, as well as the ratio of the volume of the anterior part of the swimbladder to the total swimbladder volume. Finally, we determined the ratio of the sagitta length to the fish standard length.

### Ethical statement

All procedures and all methods were approved by the ethical commission of the University of Liège (ethics case 1532). All experiments were performed in accordance with the relevant guidelines and regulations.

### Statistical analysis

The variables were tested for the assumption of normality using Kolmogorov-Smirnov tests. A nested ANOVA was performed to compare sound pressure thresholds (dependent variable) obtained at the different frequencies tested. Species and frequency were selected as fixed factors. Tukey’s HSD *post hoc* tests were performed to compare sound pressure thresholds between species at each frequency.

Linear regressions were used to examine the relationship between fish size and auditory sensitivity at the different frequencies and also to determine if there was a relationship between the averaged morphological measurements of the different auditory structures and auditory sensitivity at the different frequencies. We also looked for trends between morphological features and auditory sensitivity at three larger frequency bands (50–1200 Hz, 1500–2400 Hz and 2700–3600 Hz).

Statistical analyses were performed with Statistica 13 and GraphPad Prism 5.0. Significance level was determined at *P* < 0.05.

### Data availability

The datasets generated and analysed during the current study are available from the corresponding author on reasonable request.

## Electronic supplementary material

Supplementary information
