# Supplementary material for: Hearing capacities and morphology of the auditory system in Serrasalmidae (Teleostei: Otophysi)
Source: Sci Rep. 2018 Jan 19;8:1281. doi: 10.1038/s41598-018-19812-1 (PMC5775314; doi:10.1038/s41598-018-19812-1)
Supplement: Supplementary file 1 — Supplementary information [file 41598_2018_19812_MOESM1_ESM.pdf]

Hearing capacities and morphology of the auditory system in Serrasalmidae

Geoffrey Mélotte[1,*], Eric Parmentier[1], Christian Michel[2], Anthony Herrel[3] and Kelly Boyle[3, 4]

[1] *Laboratoire de Morphologie Fonctionnelle et Evolutive, Institut de Chimie, Bât. B6c, Université de Liège, B-4000 Liège, Belgium*

[2] *Aquarium-Muséum, Département de Biologie, Ecologie et Evolution, Université de Liège, Institut de Zoologie, Bât I1, 22 quai Van Beneden, B - 4020 Liège, Belgium*

[3] *UMR 7179 C.N.R.S./M.N.H.N., Département Adaptations du vivant, 55 Rue Buffon, Case Postale 55, 75005, Paris Cedex 5, France*

[4] *Department of Marine Sciences, University of South Alabama, 5871 USA Drive North, Mobile, Alabama 36688, USA; and Dauphin Island Sea Lab, 101 Bienville Boulevard, Dauphin Island, Alabama 36528, USA*

[*]Author for correspondence (Geoffrey.Melotte@ulg.ac.be)

SUPPLEMENTARY INFORMATION

Table S1. Comparison of AEP thresholds at different frequencies among eight serrasalmid species.

	Frequency (Hz)	*P. piraya*	*P. brachypomus*	*M. lippincottianus*	*M. rubripinnis*	*M. schomburgkii*	*S. elongatus*	*S. spilopleura*
P. brachypomus	50	NS	–	–	–	–	–	–
	150	NS	–	–	–	–	–	–
	300	0.0015	–	–	–	–	–	–
	600	NS	–	–	–	–	–	–
	900	0.025	–	–	–	–	–	–
	1200	NS	–	–	–	–	–	–
	1500	NS	–	–	–	–	–	–
	1800	NS	–	–	–	–	–	–
	2100	< 0.0001	–	–	–	–	–	–
	2400	NS	–	–	–	–	–	–
	2700	NS	–	–	–	–	–	–
	3000	NS	–	–	–	–	–	–
	3300	–	–	–	–	–	–	–
	3600	–	–	–	–	–	–	–
M. lippincottianus	50	NS	NS	–	–	–	–	–
	150	NS	NS	–	–	–	–	–
	300	NS	NS	–	–	–	–	–
	600	NS	NS	–	–	–	–	–
	900	NS	NS	–	–	–	–	–
	1200	NS	< 0.0001	–	–	–	–	–
	1500	NS	0.041	–	–	–	–	–
	1800	NS	NS	–	–	–	–	–
	2100	< 0.0001	NS	–	–	–	–	–
	2400	NS	NS	–	–	–	–	–
	2700	NS	NS	–	–	–	–	–
	3000	NS	NS	–	–	–	–	–
	3300	–	NS	–	–	–	–	–
	3600	–	NS	–	–	–	–	–
M. rubripinnis	50	NS	NS	NS	–	–	–	–
	150	NS	NS	NS	–	–	–	–
	300	NS	NS	NS	–	–	–	–
	600	NS	NS	NS	–	–	–	–
	900	NS	NS	NS	–	–	–	–
	1200	NS	NS	NS	–	–	–	–
	1500	NS	NS	NS	–	–	–	–

	1800	NS	NS	NS	–	–	–	–
	2100	NS	NS	< 0.0001	–	–	–	–
	2400	NS	NS	NS	–	–	–	–
	2700	NS	NS	NS	–	–	–	–
	3000	NS	NS	NS	–	–	–	–
	3300	–	–	–	–	–	–	–
	3600	–	–	–	–	–	–	–
M. schomburgkii	50	NS	NS	NS	NS	–	–	–
	150	NS	NS	NS	NS	–	–	–
	300	NS	NS	NS	NS	–	–	–
	600	NS	NS	NS	NS	–	–	–
	900	NS	NS	NS	NS	–	–	–
	1200	NS	< 0.0001	NS	NS	–	–	–
	1500	NS	< 0.0001	NS	NS	–	–	–
	1800	NS	0.0002	NS	NS	–	–	–
	2100	< 0.0001	NS	NS	NS	–	–	–
	2400	NS	< 0.0001	< 0.0001	NS	–	–	–
	2700	NS	NS	0.018	NS	–	–	–
	3000	NS	NS	NS	NS	–	–	–
	3300	–	NS	NS	–	–	–	–
	3600	–	NS	NS	–	–	–	–
S. elongatus	50	NS	NS	NS	NS	NS	–	–
	150	NS	NS	NS	NS	NS	–	–
	300	NS	NS	NS	NS	NS	–	–
	600	NS	NS	NS	NS	NS	–	–
	900	NS	0.010	NS	NS	NS	–	–
	1200	NS	0.0002	NS	NS	NS	–	–
	1500	NS	0.0009	NS	NS	NS	–	–
	1800	NS	NS	NS	NS	NS	–	–
	2100	< 0.0001	NS	NS	< 0.0001	NS	–	–
	2400	NS	NS	NS	NS	NS	–	–
	2700	NS	NS	NS	NS	NS	–	–
	3000	NS	NS	NS	NS	NS	–	–
	3300	–	NS	NS	–	NS	–	–
	3600	–	–	–	–	–	–	–
S. spilopleura	50	NS	NS	NS	NS	NS	NS	–
	150	NS	NS	NS	NS	NS	NS	–
	300	NS	NS	NS	NS	NS	NS	–
	600	NS	NS	NS	NS	NS	NS	–
	900	NS	0.018	NS	NS	NS	NS	–
	1200	NS	< 0.0001	NS	NS	NS	NS	–
	1500	NS	0.016	NS	NS	NS	NS	–
	1800	NS	NS	NS	NS	NS	NS	–

	2100	< 0.0001	NS	NS	< 0.0001	NS	NS	–
	2400	NS	< 0.0001	< 0.0001	NS	NS	NS	–
	2700	NS	NS	NS	NS	NS	NS	–
	3000	NS	NS	NS	NS	NS	NS	–
	3300	–	NS	NS	–	NS	NS	–
	3600	–	NS	NS	–	NS	–	–
P. nattereri	50	NS	NS	NS	NS	NS	NS	NS
	150	NS	NS	NS	NS	NS	NS	NS
	300	NS	< 0.0001	NS	NS	NS	NS	NS
	600	NS	NS	0.007	NS	NS	NS	NS
	900	NS	< 0.0001	0.017	NS	NS	NS	NS
	1200	NS	< 0.0001	NS	NS	NS	NS	NS
	1500	NS	< 0.0001	NS	NS	NS	NS	NS
	1800	NS	0.0003	NS	NS	NS	NS	NS
	2100	< 0.0001	0.001	NS	< 0.0001	NS	NS	NS
	2400	NS	< 0.0001	0.004	NS	NS	NS	NS
	2700	NS	NS	0.003	NS	NS	NS	NS
	3000	NS	NS	NS	NS	NS	NS	NS
	3300	–	NS	NS	–	NS	NS	NS
	3600	–	NS	NS	–	NS	–	NS

NS, Non-Significant differences. Results refer to nested ANOVA with Tukey’s multiple comparisons.

Table S2. Results of the linear regressions of fish standard length against hearing thresholds at the different frequencies**.**

	50 Hz			150 Hz			300 Hz			600 Hz			900 Hz		
Species	Equation	R^2	*P*-value	Equation	R^2	*P*-value	Equation	R^2	*P*-value	Equation	R^2	*P*-value	Equation	R^2	*P*-value
P. brachypomus	$y = -0.202x + 112.9$	0.026	0.657	$y = 0.140x + 83.2$	0.037	0.593	$y = -0.042x + 107.6$	0.002	0.891	$y = -0.235x + 109.4$	0.236	0.155	$y = 0.343x + 80.6$	0.218	0.173
M. lippincottianus	$y = -0.806x + 163.2$	0.103	0.366	$y = -0.496x + 131$	0.304	0.099	$y = -0.258x + 115.2$	0.057	0.505	$y = 0.213x + 75.1$	0.019	0.702	$y = -0.705x + 158.9$	0.098	0.380
M. rubripinnis	$y = 0.395x + 53.2$	0.047	0.861	$y = 1.053x – 11.6$	0.609	0.430	$y = -0.026x + 94.1$	0.002	0.975	$y = 0.474x + 35.1$	0.158	0.740	$y = 0.132x + 78.7$	0.047	0.861
M. schomburgkii	$y = -0.377x + 114.7$	0.031	0.675	$y = 0.094x + 87.6$	0.002	0.911	$y = -0.871x + 145.9$	0.063	0.549	$y = 0.447x + 61.5$	0.202	0.264	$y = -0.408x + 120.5$	0.055	0.575
S. elongatus	$y = 0.271x + 81.1$	0.102	0.601	$y = 0.281x + 66.4$	0.051	0.714	$y = -0.322x + 116.9$	0.072	0.664	$y = 1.289x – 5.8$	0.854	**0.025**	$y = 0.181x + 77.6$	0.044	0.734
S. spilopleura	$y = -0.157x + 101$	0.216	0.294	$y = 0.143x + 76.9$	0.178	0.346	$y = 0.03x + 89.6$	0.002	0.925	$y = 0.138x + 69.5$	0.083	0.530	$y = 0.019x + 92$	0.002	0.926
P. nattereri	$y = 2.153x – 46.1$	0.328	0.084	$y = 1.259x + 0.99$	0.217	0.175	$y = 1.471x – 15.1$	0.078	0.435	$y = 0.294x + 59.7$	0.011	0.777	$y = -1.118x + 163.6$	0.056	0.509
P. piraya	$y = -0.481x + 140.7$	0.634	0.107	$y = -0.458x + 143.5$	0.079	0.647	$y = 0.076x + 76.7$	0.005	0.907	$y = 0.573x + 27.6$	0.835	**0.030**	$y = 1.232x – 37.7$	0.722	0.069

R^2, coefficient of determination; *P*-value in bold are significant.

	1200 Hz			1500 Hz			1800 Hz			2100 Hz			2400 Hz		
Species	Equation	R^2	*P*-value	Equation	R^2	*P*-value	Equation	R^2	*P*-value	Equation	R^2	*P*-value	Equation	R^2	*P*-value
P. brachypomus	$y = 0.056x + 115.5$	0.011	0.771	$y = 0.522x + 73.2$	0.522	**0.018**	$y = 0.404x + 86.8$	0.362	0.066	$y = 0.227x + 103.9$	0.179	0.224	$y = -0.152x + 145.8$	0.025	0.735
M. lippincottianus	$y = 0.067x + 97.9$	0.003	0.872	$y = 0.437x + 68.3$	0.139	0.289	$y = -0.224x + 131.4$	0.056	0.511	$y = 0.328x + 86.3$	0.071	0.456	$y = 0.411x + 98.3$	0.053	0.618
M. rubripinnis	$y = 0.211x + 77.6$	0.842	0.260	$y = -0.947x + 195.9$	0.842	0.260	$y = -0.026x + 114.1$	0.002	0.974	$y = 0.474x + 93.1$	0.158	0.740	$y = -0.211x + 142.4$	0.842	0.260
M. schomburgkii	$y = -0.149x + 108.8$	0.006	0.856	$y = 0.235x + 79.9$	0.011	0.805	$y = 0.792x + 57.1$	0.121	0.399	$y = 0.929x + 67.3$	0.067	0.537	$y = 0.377x + 91.8$	0.033	0.669
S. elongatus	$y = -0.488x + 133.3$	0.194	0.458	$y = -1.228x + 182.3$	0.784	**0.046**	$y = -0.859x + 169.9$	0.959	**0.004**	$y = -0.595x + 155.4$	0.135	0.543	$y = -0.820x + 180.9$	0.337	0.305
S. spilopleura	$y = 0.081x + 89.4$	0.020	0.761	$y = -0.136x + 112.3$	0.057	0.605	$y = 0.052x + 108.2$	0.014	0.798	$y = 0.527x + 68.5$	0.563	0.052	$y = 0.036x + 108.7$	0.023	0.744
P. nattereri	$y = 0.777x + 40.8$	0.176	0.228	$y = 0.918x + 32.4$	0.194	0.203	$y = 1.671x – 6.3$	0.163	0.248	$y = -0.541x + 144.2$	0.031	0.625	$y = -0.059x + 119.2$	0.001	0.941
P. piraya	$y = -0.301x + 136.9$	0.159	0.507	$y = 0.363x + 68.9$	0.287	0.352	$y = 0.072x + 100.9$	0.013	0.854	$y = -1.178x + 267.9$	0.803	**0.040**	$y = 0.746x + 44$	0.601	0.124

R^2, coefficient of determination; *P*-value in bold are significant.

	2700 Hz			3000 Hz			3300 Hz			3600 Hz		
Species	Equation	R^2	*P*-value	Equation	R^2	*P*-value	Equation	R^2	*P*-value	Equation	R^2	*P*-value
P. brachypomus	$y = -0.340x + 146.1$	0.202	0.551	$y = 0.296x + 91.4$	0.081	0.494	/	/	/	/	/	/
M. lippincottianus	$y = -0.093x + 128.7$	0.029	0.689	$y = 0.073x + 109.9$	0.002	0.909	$y = -0.633x + 166.3$	0.107	0.527	/	/	/
M. rubripinnis	$y = -1.00x + 220$	0.571	0.454	/	/	/	/	/	/	/	/	/
M. schomburgkii	$y = -0.494x + 135.7$	0.138	0.365	$y = 1.257x + 39.3$	0.569	**0.031**	$y = -0.165x + 121.7$	0.009	0.828	$y = -0.266x + 127.2$	0.332	0.309
S. elongatus	$y = -1.253x + 206.1$	0.346	0.297	$y = -1.119x + 197.4$	0.816	**0.036**	$y = -0.704x + 172.1$	0.505	0.179	/	/	/
S. spilopleura	$y = 0.196x + 92.3$	0.203	0.311	$y = 0.289x + 83.4$	0.341	0.169	$y = 0.224x + 91.3$	0.224	0.284	/	/	/
P. nattereri	$y = 1.294x + 19.9$	0.164	0.246	$y = 1.224x + 25$	0.119	0.330	$y = 1.729x – 4.3$	0.548	**0.014**	$y = -0.5x + 146.5$	0.222	0.529
P. piraya	$y = 0.332x + 77.5$	0.083	0.639	/	/	/	/	/	/	/	/	/

R^2, coefficient of determination; *P*-value in bold are significant.

Table S3. Absolute and relative morphological measures of otoliths in *Pygocentrus nattereri*, *Myloplus rubripinnis*, *Myleus schomburgkii*, *Piaractus brachypomus*, *Serrasalmus elongatus* and *Metynnis lippincottianus*.

		Lapillus				Sagitta						Asteriscus			
	SL (mm)	V (mm^3)	V/Vot (%)	S (mm^2)	S/Sot (%)	V (mm^3)	V/Vot (%)	S (mm^2)	S/Sot (%)	L (mm)	L/SL (%)	V (mm^3)	V/Vot (%)	S (mm^2)	S/Sot (%)
P. nattereri A	120	4.4	38.3	17.4	33.9	0.4	3.4	5.3	10.4	4.2	3.5	6.7	58.4	28.6	55.7
P. nattereri B	155	5.9	39.5	20.7	35.5	0.3	1.7	3.9	6.7	4.5	2.9	8.7	58.8	33.6	57.8
P. nattereri C	134	5.5	39.3	18.9	33.4	0.3	1.8	4.2	7.3	4.0	3.0	8.3	58.8	33.6	59.2
Mean values	136	5.3	39.0	19.0	34.3	0.3	2.3	4.5	8.1	4.2	3.1	7.9	58.7	31.9	57.6
M. rubripinnis A	140	4.0	29.0	15.9	28.3	0.6	4.6	7.4	13.2	5.1	3.7	9.2	66.3	33.0	58.5
M. rubripinnis B	136	3.2	29.2	13.7	28.5	0.5	4.7	6.1	12.6	4.5	3.3	7.3	66.1	28.3	58.9
Mean values	138	3.6	29.1	14.8	28.4	0.55	4.7	6.8	12.9	4.8	3.5	8.3	66.2	30.7	58.7
M. schomburgkii A	252	7.6	26.6	27.0	24.9	1.0	3.5	10.6	9.7	6.2	2.5	21.1	70.9	70.8	65.4
M. schomburgkii B	242	8.6	21.6	27.9	22.4	1.9	4.8	15.8	12.7	7.0	2.9	29.5	73.6	80.9	64.9
Mean values	247	8.1	23.6	27.5	23.7	1.5	4.2	13.2	11.2	6.6	2.7	25.3	72.2	75.9	65.1
P. brachypomus A	139	1.8	25.2	9.8	23.6	0.5	7.2	7.3	17.6	6.1	4.4	4.8	67.6	24.5	58.8
P. brachypomus B	168	2.2	22.0	10.9	21.3	0.7	6.7	8.6	16.7	6.1	3.6	7.0	71.3	31.7	61.9
P. brachypomus C	131	1.3	22.8	8.3	21.0	0.4	6.7	6.7	16.9	5.8	4.4	4.1	70.5	24.5	62.1
Mean values	146	1.8	23.3	9.7	22.0	0.5	6.9	7.5	17.1	6.0	4.1	5.3	69.8	26.9	60.9
S. elongatus A	119	3.3	20.7	13.1	21.5	0.8	5.2	9.3	15.3	6.1	5.2	11.7	74.0	38.5	63.3
S. elongatus B	152	0.6	16.4	4.7	19.0	0.2	5.2	3.6	14.5	4.0	2.6	3.1	78.4	16.5	66.6
S. elongatus C	88	0.3	18.6	3.0	21.0	0.1	5.8	2.3	16.1	3.4	3.8	1.4	75.6	9.0	62.9
S. elongatus D	63	0.2	21.0	2.5	27.9	0.1	8.2	1.6	18.5	2.5	3.9	0.6	70.8	4.7	53.6
Mean values	105.5	1.1	19.2	5.8	22.3	0.3	6.1	4.2	16.1	4.0	3.9	4.2	74.7	17.2	61.6
M. lippincottianus A	102	1.0	15.7	6.3	19.4	0.4	6.2	5.5	17.1	3.9	3.8	4.8	78.1	20.6	63.6

M. lippincottianus B	96	1.0	15.9	6.2	19.1	0.3	5.4	5.0	15.4	4.4	4.6	5.0	78.6	21.3	65.4
M. lippincottianus C	112	0.2	1.8	8.5	18.3	0.5	4.7	8.6	18.6	4.6	4.1	9.1	93.5	29.3	63.1
M. lippincottianus D	109	0.5	4.6	8.3	16.8	0.3	2.8	7.3	14.7	5.2	4.7	10.8	92.6	34.0	68.5
Mean values	105	0.7	9.5	7.3	18.4	0.4	4.8	6.6	16.5	4.5	4.3	7.4	85.7	26.3	65.2

SL, standard length; V, volume of the otolith; V/Vot, volume of the otolith divided by total otolith volume; S, surface area of the otolith; S/Sot, surface area of the otolith divided by total otolith surface area; L, length of the sagitta; L/SL, length of the sagitta divided by standard length of the fish.

Table S4. Absolute and relative morphological measures of Weberian ossicles and the anterior part of the swimbladder in *Pygocentrus nattereri*, *Myloplus rubripinnis*, *Myleus schomburgkii*, *Piaractus brachypomus*, *Serrasalmus elongatus* and *Metynnis lippincottianus*.

		Tripus		Intercalarium		Scaphium		Ant. bladder	
	SL (mm)	V (mm^3)	V/Vwo (%)	V (mm^3)	V/Vwo (%)	V (mm^3)	V/Vwo (%)	V (mm^3)	V/Vsb (%)
P. nattereri A	120	3.9	78.5	0.45	9.1	0.62	12.4	3730	89.1
P. nattereri B	155	9.4	83.2	0.59	5.2	1.31	11.6	4490	91.4
P. nattereri C	134	7.1	83.7	0.36	4.2	1.03	12.1	4947	85.4
Mean values	136	6.8	81.8	0.47	6.1	0.99	12.1	4389	88.6
M. rubripinnis A	140	3.3	75.7	0.27	6.2	0.79	18.1	3600	46.2
M. rubripinnis B	136	2.9	78.8	0.24	6.6	0.53	14.6	2775	37.4
Mean values	138	3.1	77.3	0.26	6.4	0.66	16.3	3188	41.8
M. schomburgkii A	252	31.8	82.1	1.59	4.1	5.32	13.8	18496	46.4
M. schomburgkii B	242	27.0	80.0	2.02	6.0	4.73	14.0	21193	59.2
Mean values	247	29.4	81.1	1.81	5.0	5.03	13.9	19845	52.8
P. brachypomus A	139	3.0	80.4	0.22	5.9	0.51	13.7	4068	71.7
P. brachypomus B	168	6.6	75.4	0.84	9.6	1.31	15.0	2870	64.8
P. brachypomus C	131	2.7	68.9	0.43	10.8	0.80	20.2	2790	64.1
Mean values	146	4.1	74.9	0.50	8.8	0.87	16.3	3243	66.9
S. elongatus A	119	4.9	82.4	0.28	4.7	0.77	12.9	4442	69.9
S. elongatus B	152	2.4	81.0	0.16	5.6	0.39	13.4	1351	65.4
S. elongatus C	88	0.8	76.4	0.07	6.5	0.17	17.1	631	40.8
S. elongatus D	63	0.3	72.9	0.02	6.0	0.08	21.1	176	59.6
Mean values	105.5	2.1	78.2	0.13	5.7	0.35	16.1	1650	58.9
M. lippincottianus A	102	1.5	79.7	0.08	4.1	0.31	16.2	798	28.3
M. lippincottianus B	96	1.0	74.4	0.09	7.1	0.24	18.4	627	31.3
M. lippincottianus C	112	1.2	70.1	0.07	3.9	0.44	26.0	1233	/
M. lippincottianus D	109	1.3	69.9	0.10	5.2	0.48	24.9	1551	/
Mean values	105	1.3	73.5	0.09	5.1	0.37	21.4	1052	29.8

SL, standard length; V, volume of the Weberian ossicle; V/Vwo, volume of the Weberian ossicle divided by total Weberian ossicle volume; V/Vsb, volume of the anterior part of the swimbladder divided by total swimbladder volume.

Table S5. Results of the linear regressions of hearing structures (otoliths + Weberian ossicles) morphology against hearing thresholds at the different frequencies**.**

**Tripus (relative volume)**	50 Hz			150 Hz			300 Hz			600 Hz			900 Hz		
	Equation	R^2	*P*-value	Equation	R^2	*P*-value	Equation	R^2	*P*-value	Equation	R^2	*P*-value	Equation	R^2	*P*-value
Serrasalmidae	$y = -23x + 114.3$	0.083	0.580	$y = -62.1x + 139.4$	0.177	0.407	$y = -127.5x + 193$	0.364	0.205	$y = -113.8x + 175$	0.643	0.055	$y = -157x + 218.7$	0.479	0.128

	1200 Hz			1500 Hz			1800 Hz			2100 Hz			2400 Hz		
	Equation	R^2	*P*-value	Equation	R^2	*P*-value	Equation	R^2	*P*-value	Equation	R^2	*P*-value	Equation	R^2	*P*-value
Serrasalmidae	$y = -187.7x + 248.4$	0.436	0.153	$y = -202.5x + 257.5$	0.607	0.068	$y = -161.5x + 235.8$	0.768	**0.022**	$y = -62.8x + 168.8$	0.030	0.745	$y = -236.2x + 306.7$	0.88	**0.006**

	2700 Hz			3000 Hz			3300 Hz			3600 Hz		
	Equation	R^2	*P*-value	Equation	R^2	*P*-value	Equation	R^2	*P*-value	Equation	R^2	*P*-value
Serrasalmidae	$y = -176.9x + 252$	0.876	**0.006**	$y = -82.3x + 177.1$	0.421	0.163	$y = -65.1x + 166.5$	0.270	0.37	$y = -83.4x + 180.9$	0.804	0.103

R^2, coefficient of determination; *P*-value in bold are significant.

**Intercalarium (relative volume)**	50 Hz			150 Hz			300 Hz			600 Hz			900 Hz		
	Equation	R^2	*P*-value	Equation	R^2	*P*-value	Equation	R^2	*P*-value	Equation	R^2	*P*-value	Equation	R^2	*P*-value
Serrasalmidae	$y = 0.502x + 96.4$	< 0.0001	0.996	$y = 116.1x + 83.9$	0.110	0.520	$y = 247x + 78.5$	0.244	0.320	$y = 33.2x + 84.4$	0.010	0.852	$y = 282.2x + 79.1$	0.276	0.285

	1200 Hz			1500 Hz			1800 Hz			2100 Hz			2400 Hz		
	Equation	R^2	*P*-value	Equation	R^2	*P*-value	Equation	R^2	*P*-value	Equation	R^2	*P*-value	Equation	R^2	*P*-value
Serrasalmidae	$y = 505.6x + 71$	0.565	0.085	$y = 477x + 70.4$	0.601	0.070	$y = 315.6x + 90.6$	0.524	0.104	$y = 222x + 106.2$	0.066	0.623	$y = 317.7x + 103.3$	0.284	0.276

	2700 Hz			3000 Hz			3300 Hz			3600 Hz		
	Equation	R^2	*P*-value	Equation	R^2	*P*-value	Equation	R^2	*P*-value	Equation	R^2	*P*-value
Serrasalmidae	$y = 94.2x + 108.6$	0.044	0.689	$y = 4.42x + 112.8$	0.0002	0.978	$y = 53.5x + 112.5$	0.032	0.772	$y = 152.2x + 106.5$	0.466	0.318

R^2, coefficient of determination; *P*-value in bold are significant.

**Scaphium (relative volume)**	50 Hz			150 Hz			300 Hz			600 Hz			900 Hz		
	Equation	R^2	*P*-value	Equation	R^2	*P*-value	Equation	R^2	*P*-value	Equation	R^2	*P*-value	Equation	R^2	*P*-value
Serrasalmidae	$y = 24.9x + 92.5$	0.088	0.568	$y = 45.5x + 83.8$	0.086	0.573	$y = 91.4x + 79.1$	0.169	0.418	$y = 118.5x + 67.4$	0.629	0.060	$y = 117.2x + 77.8$	0.241	0.323

	1200 Hz			1500 Hz			1800 Hz			2100 Hz			2400 Hz		
	Equation	R^2	*P*-value	Equation	R^2	*P*-value	Equation	R^2	*P*-value	Equation	R^2	*P*-value	Equation	R^2	*P*-value
Serrasalmidae	$y = 106.7x + 85.3$	0.128	0.487	$y = 128.7x + 79.4$	0.221	0.346	$y = 115.2x + 91.7$	0.353	0.214	$y = 25.4x + 115.9$	0.004	0.901	$y = 196.8x + 91.4$	0.552	0.091

	2700 Hz			3000 Hz			3300 Hz			3600 Hz		
	Equation	R^2	*P*-value	Equation	R^2	*P*-value	Equation	R^2	*P*-value	Equation	R^2	*P*-value
Serrasalmidae	$y = 175.4x + 86.3$	0.778	**0.02**	$y = 89.2x + 98.8$	0.447	0.147	$y = 60.3x + 106.2$	0.210	0.438	$y = 62x + 106.1$	0.404	0.364

R^2, coefficient of determination; *P*-value in bold are significant.

**Sagitta (relative length)**	50 Hz			150 Hz			300 Hz			600 Hz			900 Hz		
	Equation	R^2	*P*-value	Equation	R^2	*P*-value	Equation	R^2	*P*-value	Equation	R^2	*P*-value	Equation	R^2	*P*-value
Serrasalmidae	$y = 270.6x + 86.7$	0.415	0.167	$y = 44.9x + 89.5$	0.003	0.914	$y = 511x + 75.4$	0.211	0.360	$y = 455.3x + 70$	0.371	0.199	$y = 614.8x + 74.4$	0.265	0.296

	1200 Hz			1500 Hz			1800 Hz			2100 Hz			2400 Hz		
	Equation	R^2	*P*-value	Equation	R^2	*P*-value	Equation	R^2	*P*-value	Equation	R^2	*P*-value	Equation	R^2	*P*-value
Serrasalmidae	$y = 829.3x + 72.5$	0.307	0.254	$y = 977.9x + 64.7$	0.511	0.111	$y = 828.6x + 80.3$	0.730	**0.03**	$y = -190.6x + 126.8$	0.010	0.852	$y = 1247x + 78$	0.885	**0.005**

	2700 Hz			3000 Hz			3300 Hz			3600 Hz		
	Equation	R^2	*P*-value	Equation	R^2	*P*-value	Equation	R^2	*P*-value	Equation	R^2	*P*-value
Serrasalmidae	$y = 908.8x + 81.7$	0.834	**0.011**	$y = 460.3x + 96.5$	0.475	0.130	$y = 445.2x + 99.6$	0.452	0.214	$y = 469.9x + 99.3$	0.878	0.063

R^2, coefficient of determination; *P*-value in bold are significant.

**Sagitta (relative volume)**	50 Hz			150 Hz			300 Hz			600 Hz			900 Hz		
	Equation	R^2	*P*-value	Equation	R^2	*P*-value	Equation	R^2	*P*-value	Equation	R^2	*P*-value	Equation	R^2	*P*-value
Serrasalmidae	$y = 46.7x + 94.2$	0.080	0.587	$y = 116.7x + 85.5$	0.146	0.455	$y = 385.6x + 75.2$	0.777	**0.02**	$y = 190.5x + 77.3$	0.420	0.164	$y = 275.5x + 83.3$	0.344	0.221

	1200 Hz			1500 Hz			1800 Hz			2100 Hz			2400 Hz		
	Equation	R^2	*P*-value	Equation	R^2	*P*-value	Equation	R^2	*P*-value	Equation	R^2	*P*-value	Equation	R^2	*P*-value
Serrasalmidae	$y = 460.4x + 80.2$	0.613	0.066	$y = 372.8x + 82$	0.48	0.127	$y = 285.9x + 96.4$	0.562	0.086	$y = 175.3x + 111.5$	0.054	0.659	$y = 382.1x + 104.6$	0.537	0.097

	2700 Hz			3000 Hz			3300 Hz			3600 Hz		
	Equation	R^2	*P*-value	Equation	R^2	*P*-value	Equation	R^2	*P*-value	Equation	R^2	*P*-value
Serrasalmidae	$y = 288.9x + 100.5$	0.545	0.094	$y = 223.9x + 102.3$	0.727	**0.031**	$y = 215.3x + 105.4$	0.690	0.081	$y = 170x + 108.3$	0.659	0.188

R^2, coefficient of determination; *P*-value in bold are significant.

**Sagitta (relative surface area)**	50 Hz			150 Hz			300 Hz			600 Hz			900 Hz		
	Equation	R^2	*P*-value	Equation	R^2	*P*-value	Equation	R^2	*P*-value	Equation	R^2	*P*-value	Equation	R^2	*P*-value
Serrasalmidae	$y = 32x + 92.1$	0.185	0.394	$y = 41.1x + 85.5$	0.089	0.565	$y = 154.6x + 72.7$	0.617	0.064	$y = 105.8x + 72$	0.642	0.055	$y = 132.7x + 78.4$	0.395	0.182

	1200 Hz			1500 Hz			1800 Hz			2100 Hz			2400 Hz		
	Equation	R^2	*P*-value	Equation	R^2	*P*-value	Equation	R^2	*P*-value	Equation	R^2	*P*-value	Equation	R^2	*P*-value
Serrasalmidae	$y = 186.9x + 76.8$	0.500	0.116	$y = 171.2x + 76.6$	0.501	0.116	$y = 137.2x + 91.5$	0.640	0.056	$y = 29.4x + 115.9$	0.007	0.871	$y = 205x + 95$	0.765	**0.023**

	2700 Hz			3000 Hz			3300 Hz			3600 Hz		
	Equation	R^2	*P*-value	Equation	R^2	*P*-value	Equation	R^2	*P*-value	Equation	R^2	*P*-value
Serrasalmidae	$y = 160.7x + 92.5$	0.834	**0.011**	$y = 108.1x + 98.3$	0.839	**0.010**	$y = 95.5x + 102.6$	0.666	0.092	$y = 80.6x + 105.3$	0.773	0.121

R^2, coefficient of determination; *P*-value in bold are significant.

**Lapillus (relative volume)**	50 Hz			150 Hz			300 Hz			600 Hz			900 Hz		
	Equation	R^2	*P*-value	Equation	R^2	*P*-value	Equation	R^2	*P*-value	Equation	R^2	*P*-value	Equation	R^2	*P*-value
Serrasalmidae	$y = -8.34x + 98.5$	0.098	0.546	$y = -8.60x + 93.2$	0.03	0.741	$y = -40.8x + 103.5$	0.334	0.230	$y = -41.6x + 96.4$	0.768	**0.022**	$y = -38.3x + 105.7$	0.255	0.307

	1200 Hz			1500 Hz			1800 Hz			2100 Hz			2400 Hz		
	Equation	R^2	*P*-value	Equation	R^2	*P*-value	Equation	R^2	*P*-value	Equation	R^2	*P*-value	Equation	R^2	*P*-value
Serrasalmidae	$y = -35.7x + 110.9$	0.141	0.463	$y = -30.9x + 107.4$	0.127	0.488	$y = -24.7x + 116.1$	0.161	0.431	$y = 14x + 116.6$	0.013	0.829	$y = -51.3x + 135.2$	0.371	0.199

	2700 Hz			3000 Hz			3300 Hz			3600 Hz		
	Equation	R^2	*P*-value	Equation	R^2	*P*-value	Equation	R^2	*P*-value	Equation	R^2	*P*-value
Serrasalmidae	$y = -46.7x + 125.6$	0.546	0.094	$y = -35.6x + 121.6$	0.704	**0.037**	$y = -24.5x + 121.4$	0.321	0.320	$y = -15.6x + 119.7$	0.229	0.522

R^2, coefficient of determination; *P*-value in bold are significant.

**Lapillus (relative surface area)**	50 Hz			150 Hz			300 Hz			600 Hz			900 Hz		
	Equation	R^2	*P*-value	Equation	R^2	*P*-value	Equation	R^2	*P*-value	Equation	R^2	*P*-value	Equation	R^2	*P*-value
Serrasalmidae	$y = -11.1x + 99.2$	0.057	0.65	$y = -23.3x + 96.9$	0.074	0.603	$y = -94.5x + 117.2$	0.587	0.076	$y = -77.2x + 105.6$	0.869	**0.007**	$y = -85.5x + 117.8$	0.418	0.165

	1200 Hz			1500 Hz			1800 Hz			2100 Hz			2400 Hz		
	Equation	R^2	*P*-value	Equation	R^2	*P*-value	Equation	R^2	*P*-value	Equation	R^2	*P*-value	Equation	R^2	*P*-value
Serrasalmidae	$y = -94x + 125.7$	0.322	0.240	$y = -74.7x + 118.5$	0.243	0.321	$y = -52.1x + 123.1$	0.236	0.329	$y = 13.6x + 116.6$	0.004	0.905	$y = -96.5x + 146.9$	0.432	0.156

	2700 Hz			3000 Hz			3300 Hz			3600 Hz		
	Equation	R^2	*P*-value	Equation	R^2	*P*-value	Equation	R^2	*P*-value	Equation	R^2	*P*-value
Serrasalmidae	$y = -78x + 133.8$	0.501	0.116	$y = -64.3x + 129.1$	0.756	**0.024**	$y = -46.6x + 127$	0.369	0.277	$y = -31.9x + 123.9$	0.307	0.446

R^2, coefficient of determination; *P*-value in bold are significant.

**Asteriscus (relative volume)**	50 Hz			150 Hz			300 Hz			600 Hz			900 Hz		
	Equation	R^2	*P*-value	Equation	R^2	*P*-value	Equation	R^2	*P*-value	Equation	R^2	*P*-value	Equation	R^2	*P*-value
Serrasalmidae	$y = 8.55x + 90.4$	0.086	0.573	$y = 6.5x + 86.5$	0.015	0.820	$y = 36.6x + 67.7$	0.225	0.342	$y = 43.6x + 55.4$	0.708	**0.036**	$y = 36.9x + 70.3$	0.199	0.376

	1200 Hz			1500 Hz			1800 Hz			2100 Hz			2400 Hz		
	Equation	R^2	*P*-value	Equation	R^2	*P*-value	Equation	R^2	*P*-value	Equation	R^2	*P*-value	Equation	R^2	*P*-value
Serrasalmidae	$y = 28.1x + 82.4$	0.073	0.604	$y = 25.2x + 82$	0.07	0.611	$y = 20.5x + 95.6$	0.093	0.558	$y = -22.3x + 135.9$	0.028	0.751	$y = 49.2x + 87.9$	0.287	0.274

	2700 Hz			3000 Hz			3300 Hz			3600 Hz		
	Equation	R^2	*P*-value	Equation	R^2	*P*-value	Equation	R^2	*P*-value	Equation	R^2	*P*-value
Serrasalmidae	$y = 46.7x + 81.2$	0.458	0.14	$y = 35.5x + 87.8$	0.587	0.076	$y = 22.3x + 99.7$	0.221	0.424	$y = 13.5x + 106.3$	0.146	0.619

R^2, coefficient of determination; *P*-value in bold are significant.

**Asteriscus (relative surface area)**	50 Hz			150 Hz			300 Hz			600 Hz			900 Hz		
	Equation	R^2	*P*-value	Equation	R^2	*P*-value	Equation	R^2	*P*-value	Equation	R^2	*P*-value	Equation	R^2	*P*-value
Serrasalmidae	$y = -4.76x + 99.4$	0.003	0.914	$y = 22.8x + 77.1$	0.022	0.779	$y = 106.9x + 28$	0.236	0.328	$y = 113.8x + 16.4$	0.593	0.073	$y = 106.7x + 30.9$	0.204	0.368

	1200 Hz			1500 Hz			1800 Hz			2100 Hz			2400 Hz		
	Equation	R^2	*P*-value	Equation	R^2	*P*-value	Equation	R^2	*P*-value	Equation	R^2	*P*-value	Equation	R^2	*P*-value
Serrasalmidae	$y = 65x + 62.4$	0.048	0.676	$y = 23.6x + 85.4$	0.008	0.869	$y = -5.83x + 113.8$	0.001	0.954	$y = -81.2x + 169.9$	0.046	0.685	$y = 51.1x + 91.5$	0.038	0.711

	2700 Hz			3000 Hz			3300 Hz			3600 Hz		
	Equation	R^2	*P*-value	Equation	R^2	*P*-value	Equation	R^2	*P*-value	Equation	R^2	*P*-value
Serrasalmidae	$y = 47.5x + 85.2$	0.058	0.645	$y = 69.3x + 70.5$	0.276	0.285	$y = 19.1x + 103.9$	0.017	0.833	$y = 0.124x + 115.9$	< 0.001	0.999

R^2, coefficient of determination; *P*-value in bold are significant.

**Ant. bladder (relative volume)**	50 Hz			150 Hz			300 Hz			600 Hz			900 Hz		
	Equation	R^2	*P*-value	Equation	R^2	*P*-value	Equation	R^2	*P*-value	Equation	R^2	*P*-value	Equation	R^2	*P*-value
Serrasalmidae	$y = 1.94x + 95.4$	0.023	0.776	$y = -13x + 98.5$	0.301	0.26	$y = -11.2x + 100.1$	0.108	0.524	$y = -15.1x + 94.9$	0.435	0.154	$y = -9.7x + 102$	0.07	0.611

	1200 Hz			1500 Hz			1800 Hz			2100 Hz			2400 Hz		
	Equation	R^2	*P*-value	Equation	R^2	*P*-value	Equation	R^2	*P*-value	Equation	R^2	*P*-value	Equation	R^2	*P*-value
Serrasalmidae	$y = -5.62x + 105.5$	0.015	0.817	$y = -5.1x + 102.8$	0.015	0.82	$y = -7.15x + 114.2$	0.058	0.646	$y = -24.3x + 133.7$	0.171	0.415	$y = -13.4x + 130.5$	0.109	0.524

	2700 Hz			3000 Hz			3300 Hz			3600 Hz		
	Equation	R^2	*P*-value	Equation	R^2	*P*-value	Equation	R^2	*P*-value	Equation	R^2	*P*-value
Serrasalmidae	$y = -19.7x + 125.6$	0.42	0.164	$y = -10.8x + 119.2$	0.279	0.281	$y = -6.69x + 119.8$	0.096	0.611	$y = -3.94x + 118.3$	0.061	0.753

R^2, coefficient of determination; *P*-value in bold are significant.

Table S6. Results of the linear regressions of hearing structures (otoliths + Weberian ossicles) morphology against mean hearing thresholds at the different frequency bands**.**

**Tripus (relative volume)**	Band 1 (50 - 1200 Hz)			Band 2 (1500 - 2400 Hz)			Band 3 (2700 - 3600 Hz)		
	Equation	R^2	*P*-value	Equation	R^2	*P*-value	Equation	R^2	*P*-value
Serrasalmidae	$y = -132.6x + 199.8$	0.473	0.131	$y = -183.9x + 262.2$	0.508	0.112	$y = -98.9x + 192$	0.633	0.059

R^2, coefficient of determination; *P*-value in bold are significant.

**Intercalarium (relative volume)**	Band 1 (50 - 1200 Hz)			Band 2 (1500 - 2400 Hz)			Band 3 (2700 - 3600 Hz)		
	Equation	R^2	*P*-value	Equation	R^2	*P*-value	Equation	R^2	*P*-value
Serrasalmidae	$y = 327.3x + 76.4$	0.514	0.109	$y = 285.3x + 101.4$	0.218	0.351	$y = 68.1x + 110.9$	0.054	0.659

R^2, coefficient of determination; *P*-value in bold are significant.

**Scaphium (relative volume)**	Band 1 (50 - 1200 Hz)			Band 2 (1500 - 2400 Hz)			Band 3 (2700 - 3600 Hz)		
	Equation	R^2	*P*-value	Equation	R^2	*P*-value	Equation	R^2	*P*-value
Serrasalmidae	$y = 81.3x + 83.6$	0.160	0.431	$y = 145.9x + 95.7$	0.289	0.272	$y = 94.9x + 99.9$	0.526	0.103

R^2, coefficient of determination; *P*-value in bold are significant.

**Sagitta (relative length)**	Band 1 (50 - 1200 Hz)			Band 2 (1500 - 2400 Hz)			Band 3 (2700 - 3600 Hz)		
	Equation	R^2	*P*-value	Equation	R^2	*P*-value	Equation	R^2	*P*-value
Serrasalmidae	$y = 588.5x + 75.5$	0.336	0.228	$y = 675.1x + 94.8$	0.247	0.316	$y = 578.2x + 94.3$	0.781	**0.0195**

R^2, coefficient of determination; *P*-value in bold are significant.

**Sagitta (relative volume)**	Band 1 (50 - 1200 Hz)			Band 2 (1500 - 2400 Hz)			Band 3 (2700 - 3600 Hz)		
	Equation	R^2	*P*-value	Equation	R^2	*P*-value	Equation	R^2	*P*-value
Serrasalmidae	$y = 313.4x + 81.6$	0.616	0.064	$y = 294.9x + 104.9$	0.305	0.256	$y = 221.1x + 104.5$	0.739	**0.028**

R^2, coefficient of determination; *P*-value in bold are significant.

**Sagitta (relative surface area)**	Band 1 (50 - 1200 Hz)			Band 2 (1500 - 2400 Hz)			Band 3 (2700 - 3600 Hz)		
	Equation	R^2	*P*-value	Equation	R^2	*P*-value	Equation	R^2	*P*-value
Serrasalmidae	$y = 129.9x + 78.9$	0.531	0.101	$y = 133.3x + 100.9$	0.312	0.249	$y = 109.9x + 100.1$	0.914	**0.003**

R^2, coefficient of determination; *P*-value in bold are significant.

**Lapillus (relative volume)**	Band 1 (50 - 1200 Hz)			Band 2 (1500 - 2400 Hz)			Band 3 (2700 - 3600 Hz)		
	Equation	R^2	*P*-value	Equation	R^2	*P*-value	Equation	R^2	*P*-value
Serrasalmidae	$y = -27.4x + 103.2$	0.180	0.401	$y = -23.2x + 124.6$	0.072	0.607	$y = -30.6x + 122.5$	0.543	0.095

R^2, coefficient of determination; *P*-value in bold are significant.

**Lapillus (relative surface area)**	Band 1 (50 - 1200 Hz)			Band 2 (1500 - 2400 Hz)			Band 3 (2700 - 3600 Hz)		
	Equation	R^2	*P*-value	Equation	R^2	*P*-value	Equation	R^2	*P*-value
Serrasalmidae	$y = -68.4x + 113.6$	0.370	0.200	$y = -46.2x + 130.6$	0.094	0.554	$y = -54.5x + 128.7$	0.565	0.085

R^2, coefficient of determination; *P*-value in bold are significant.

**Asteriscus (relative volume)**	Band 1 (50 - 1200 Hz)			Band 2 (1500 - 2400 Hz)			Band 3 (2700 - 3600 Hz)		
	Equation	R^2	*P*-value	Equation	R^2	*P*-value	Equation	R^2	*P*-value
Serrasalmidae	$y = 22.8x + 80.4$	0.105	0.531	$y = 18.3x + 106$	0.038	0.712	$y = 29.7x + 94$	0.427	0.159

R^2, coefficient of determination; *P*-value in bold are significant.

**Asteriscus (relative surface area)**	Band 1 (50 - 1200 Hz)			Band 2 (1500 - 2400 Hz)			Band 3 (2700 - 3600 Hz)		
	Equation	R^2	*P*-value	Equation	R^2	*P*-value	Equation	R^2	*P*-value
Serrasalmidae	$y = 53.6x + 63.7$	0.071	0.609	$y = -21.3x + 132.2$	0.006	0.881	$y = 34.9x + 93.7$	0.073	0.605

R^2, coefficient of determination; *P*-value in bold are significant.

**Ant. bladder (relative volume)**	Band 1 (50 - 1200 Hz)			Band 2 (1500 - 2400 Hz)			Band 3 (2700 - 3600 Hz)		
	Equation	R^2	*P*-value	Equation	R^2	*P*-value	Equation	R^2	*P*-value
Serrasalmidae	$y = -4.87x + 99.4$	0.025	0.767	$y = -23.5x + 132.4$	0.319	0.243	$y = -8.94x + 120.2$	0.199	0.375

R^2, coefficient of determination; *P*-value in bold are significant.
